# Clostridium difficile Infection During Palliative Capecitabine Chemotherapy: A Case Report

**DOI:** 10.7759/cureus.27102

**Published:** 2022-07-21

**Authors:** Keshav Bhattar, Palack Agrawal

**Affiliations:** 1 Internal Medicine, Dr. Sampurnanand Medical College, Jodhpur, IND; 2 Pediatrics, Joe DiMaggio Children’s Hospital, Miami, USA

**Keywords:** protein-losing enteropathy, pleural effusion, palliative chemotherapy, fidaxomicin, colitis, pseudomembranous colitis, clostridium difficile, breast cancer, capecitabine

## Abstract

Capecitabine has been used for triple-negative metastatic breast cancers both as monotherapy and in combination with other agents. However, its gastrointestinal side effects are one of the biggest challenges for its patient compliance, and often result in permanent drug withdrawal. There have been reports of it causing enterocolitis (mainly terminal ileitis) and even ischaemic colitis, but it has not frequently been directly associated with *Clostridium difficile* infection. We describe a case of a 65-year-old woman with triple-negative breast cancer on palliative capecitabine who presented with blood-streaked watery diarrhea and abdominal pain and was diagnosed with chemotherapy-induced severe colitis with superimposed *Clostridium difficile *infection.

## Introduction

Chemotherapy is usually initiated with the aim of eliminating neoplastic cells. However, when given in a non-curative setting to maximize symptom control, improve quality of life (QoL), and, ideally, to improve survival, it is known as palliative chemotherapy [1]. In patients with triple-negative breast cancer [estrogen receptor (ER) negative, progesterone receptor (PR) negative, and human epidermal growth factor receptor 2 (HER-2) negative], capecitabine, vinorelbine, gemcitabine, doxorubicin, taxanes and newer agents, such as vinflunine, irinotecan, and etirinotecan, are some of the most frequently used drugs [2, 3]. 

Capecitabine (Xeloda®, Genentech, Inc., South San Francisco, USA) is an effective agent for metastatic, receptor-negative, and advanced breast cancer, both as monotherapy or in combination with docetaxel [4]. It is a prodrug that gets enzymatically converted to its active form, fluorouracil (also called 5-fluorouracil). CRD (chemotherapy-related diarrhea) is a major dose-limiting adverse drug effect that can be fatal and necessitates hospitalization for supportive care. This is especially true for capecitabine, trials have shown that severe diarrhea (grade 3 or 4) is very common (as high as 47% in some regimens) [5]. 

## Case presentation

A 65-year-old woman on palliative capecitabine chemotherapy (14 days on, 7 days off) for triple-negative breast cancer presented to the emergency department with chief complaints of 1 week of persistent blood-streaked diarrhea. She had similar but milder symptoms at the end of the first round of capecitabine 2 weeks ago but improved during her 7 days off period. However, on resuming capecitabine during her second round of 14 days, she started to have diarrhea again.

Past history was significant for untreated chronic lymphocytic lymphoma (CLL) diagnosed 20 years ago, excision of a localized melanoma from her left medial thigh, without adjuvant treatment 13 years ago, surgical removal of a meningioma 9 years ago, and early stage, estrogen receptor-positive (ER+) breast cancer (right-sided retroareolar mass with ipsilateral axillary lymph node involvement) managed with bilateral mastectomy without any adjuvant therapy 13 years ago (refusal to chemotherapy, tamoxifen, or radiation).

Three years ago, she presented with left-sided rib and mid-thoracic back pain for two months associated with mild weight loss. Radiology revealed osseous and liver lesions consistent with metastases. She was started on palbociclib, letrozole and zolendronic acid. MRI brain showed mass at left hallux (site of previous surgery for meningioma resection) suggestive of neoplasm, and a 9 mm mass posterior to the pituitary gland. These were managed with gamma knife surgery. Meanwhile, computed tomography (CT) guided liver biopsy revealed high-grade adenocarcinoma, consistent with a breast carcinoma as the primary neoplasm. Biopsy was also ER negative, PR negative, and human epidermal growth factor receptor 2 (HER-2) immunohistochemistry 0. Guardant 360 panel showed PIK3CA (phosphatidylinositol-4,5-bisphosphate 3-kinase catalytic subunit alpha) mutation, BRCA (breast cancer gene) 1/2 negative, and PD-L1 (programmed death ligand-1 ) 0%. Because of the triple-negative status of her breast cancer, letrozole and palbociclib were stopped, and she was started on palliative paclitaxel. Eventually, paclitaxel was stopped because of severe dermatological adverse effects, and palliative docetaxel was started. Though her tumor markers (cancer antigen 27.29, cancer antigen 15-3, carcinoembryonic antigen (CEA)) continued to improve, repeat radiology revealed improvement of hepatic lesions but increasing uptake in several vertebral bodies and new lesions in bilateral femurs. She tolerated docetaxel well initially but started complaining of shortness of breath with mild exertion as well as bilateral lower limb edema. Chest x-ray showed bilateral pleural effusions, thoracocentesis revealed a transudative type of pleural effusion, and positron emission tomography & computed tomography (PET-CT) ruled out pleural-based cancer. She was started on diuretics and showed great response. Her third spacing was finally attributed to a docetaxel-induced capillary leak. She also developed grade 2 neuropathy and her volume overload worsened. She was given a 3-week chemo break to address her excess fluid, and the diuretic (spironolactone) was continued. Docetaxel was discontinued and she started palliative capecitabine therapy at 1000 mg/m^2^ twice daily x 14 days on, followed by 7 days off. However, she continued to have dyspnea due to bilateral pleural effusions and underwent thoracocentesis which resulted in the draining of 700cc of transudative fluid. 

**Figure 1:**
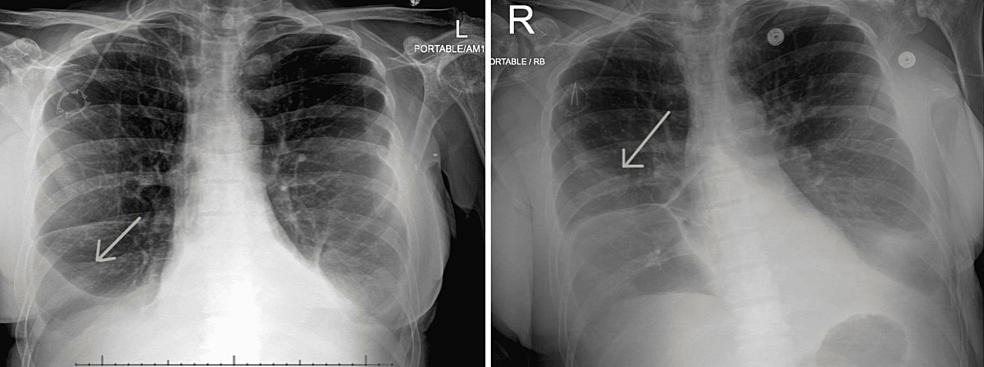
Antero-posterior chest x-ray showing mild bilateral pleural effusions on admission (LEFT) and progression of pleural effusion on day 5 of admission (RIGHT).

She reported that she had mild gastrointestinal discomfort and occasional loose stools at the end of the first round of capecitabine but improved during her 7 days off period. However, on resuming capecitabine, she presented to the emergency department with intractable watery diarrhea within the next 24 hours. The diarrhea was occasionally blood-streaked. It was associated with gradual onset cramping, epigastric abdominal pain (no guarding, rebound, or distension), anorexia, and dehydration but no nausea, vomiting, fever, chills, or dysuria. She also complained of mild dyspnea. A chest x-ray showed bilateral small-to-moderate pleural effusions with adjacent opacities, slightly improved compared to that of two weeks ago when she had her thoracocentesis (Figure 1). She was ill-appearing and cachectic but in no acute distress and was normotensive.

**Figure 2:**
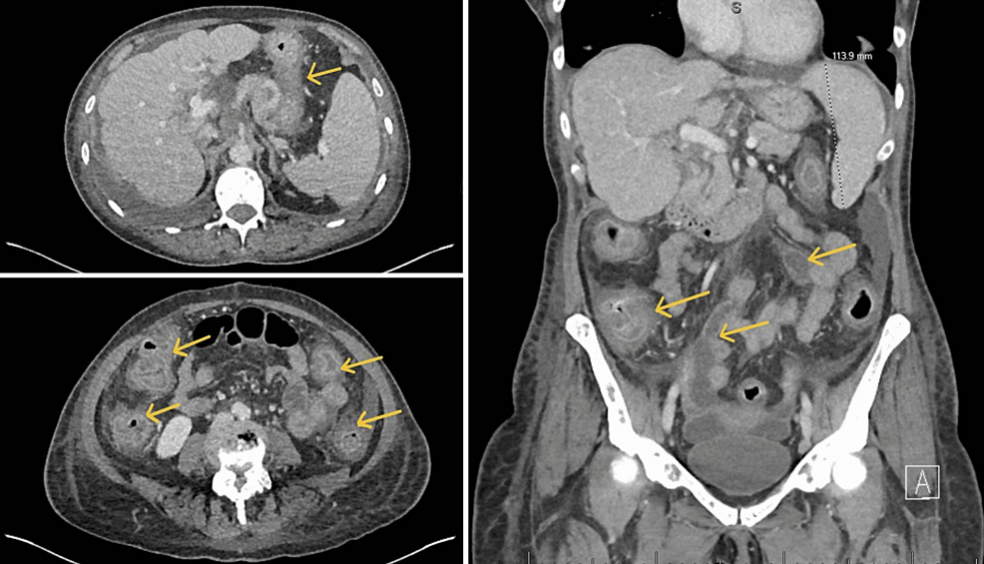
CT abdomen showing severe diffuse contiguous wall thickening of the colon suggestive of colitis (yellow arrows), mild to moderate ascites, and osseous and hepatic metastases. Multiple scattered hepatic and pelvic lesions consistent with metastatic disease.

Capecitabine was stopped and she was started on intravenous hydration with replacement of electrolytes. In view of her abdominal pain, a CT of her abdomen was ordered which showed severe diffuse contiguous wall thickening of the colon suggestive of colitis, mild to moderate ascites, osseous and hepatic metastases, and mild bilateral pleural effusions (Figure 2). Radiology advised to rule out possible *Clostridium difficile *infection (Table 1). Ova and parasite examination was also sent which came back negative. She refused to have a colonoscopy.

**Table 1:** Clostridium difficile panel ordered on the day of admission PCR - Polymerase chain reaction

Clostridium difficile panel	Results
Clostridium difficile Toxin B, Ql PCR	POSITIVE
Clostridium difficile Glutamate Dehydrogenase	POSITIVE
Clostridium difficile Toxin A/B	POSITIVE

She was started on vancomycin 125 mg orally six hourly. She continued to have watery diarrhea (10-20 times per day) in the subsequent days of her hospitalization. The metabolic panel showed hypocalcemia, hypoalbuminemia, and non-anion gap metabolic acidosis (due to persistent diarrhea) (Table 2). On day 2 of her admission, she revealed she was self-medicating with diphenoxylate/atropine for her diarrhea which was stopped immediately. She was explained that this could have led to low gastrointestinal (GI) motility, causing retention of *Clostridium difficile* infected stool in her bowel. Because of her high risk of recurrence and the severity of her colitis, she was switched to fidaxomicin (200 mg orally twice a day x 10 days) on the third day of her hospitalization. She continued to have 15-20 episodes of watery diarrhea with a progressively downtrending serum albumin level (Table 2). 

**Table 2:** Complete metabolic panel trends since admission Note the hypoalbuminemia which worsened around day 5, along with concurrent hypocalcemia (corrected calcium levels were relatively less deranged). Also note the respiratory alkalosis occurring as a consequence of the normal-anion gap metabolic acidosis. BUN - Blood urea nitrogen, AST - Aspartate transaminase, SGOT - Serum glutamic-oxaloacetic transaminase, ALT - Alanine transaminase, SGPT - Serum glutamic pyruvic transaminase, ALP - Alkaline phosphatase

PARAMETERS	REFERENCE RANGE	DAY 0	DAY 3	DAY 5	DAY 7	DAY 9
Sodium	135 - 146 mmol/L	138	138	136	142	141
Potassium	3.5 - 5.5mmol/L	4.9	3.4	3.4	4.5	4.4
Chloride	98 - 110 mmol/L	105	108	119	108	109
CO_2 _	19 - 34 mmol/L	24	18	17	26	25
Anion gap	6 - 22	9	12	10	6	7
Osmolality	275 - 295 mOsm/kg	273	273	274	281	180
BUN	8 - 23 mg/dL	8	7	4	7	11
Creatinine	0.40 - 1.10 mg/dL	0.76	0.62	0.54	0.52	0.58
Total protein	6.1 - 8.1 g/dL	4.3	3.8	2.4	4.2	-
Albumin	3.5 - 5.3 g/dL	3.0	2.7	1.7	3.0	2.9
Total calcium	8.6 - 10.3 mg/dL	7.8	7.7	5.8	8.5	7.9
Total bilirubin	0.0 - 1.2 mg/dL	1.3	1.1	0.4	0.6	-
Direct bilirubin	0.0 - 0.3 mg/dL	0.3	0.3	0.2	0.2	-
AST (SGOT)	10 - 40 U/L	67	15	11	24	-
ALT (SGPT)	0 - 33 U/L	20	13	7	14	-
ALP	35 - 130 U/L	70	75	71	110	-
Glucose	65-99 mg/dL	84	87	74	94	93
Phosphorus	2.8 - 4.5 mg/dL	-	-	2.0	3.6	3.1

**Figure 3:**
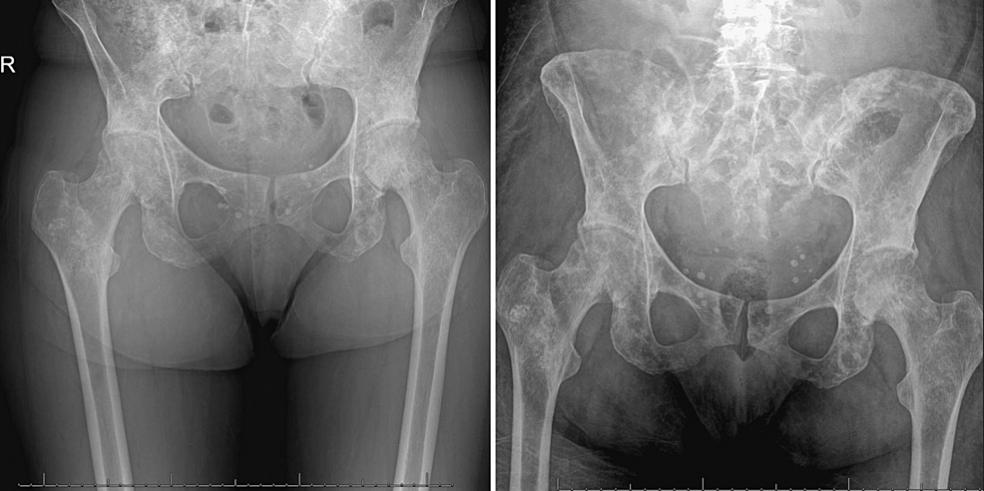
Comparative pelvic x-rays: (LEFT) Five months ago - widespread metastatic lesions in both axial and appendicular skeletal system. (RIGHT) On current admission - consistent with previous x-ray with slight disease progression, but showing no fractures

Despite multiple days of antibiotic treatment, the patient continued to have severe diarrhea resulting in severe protein malnourishment. After discussion with the team, she was started on loperamide with negligible relief. A repeat chest x-ray was done for complaints of dyspnea, wheezing, limb edema, and mild abdominal distension, and showed bilateral pleural effusions with slight progression (Figure 1). She was given intravenous furosemide and intravenous albumin supplementation for the same. She required oxygen supplementation at 3 litres per minute to maintain saturation above 94% for the next four days. Furthermore, she continued to have significant electrolyte abnormalities including hypokalemia and hypocalcemia, requiring potassium chloride and calcium gluconate, respectively. Following calcium gluconate, she developed hypophosphatemia (likely due to the calcium infusion causing increased renal excretion of phosphate) (Table 2). She also suffered a fall in the washroom. However, her orthostatic BP measurements showed no change while standing and the fall was attributed to mechanical causes (slippery surface). A pelvic x-ray revealed a diffusely heterogeneous appearance of the axial and appendicular bones consistent with known metastatic disease with no evidence of fractures (Figure 3). 

By day 9 of hospitalization, the frequency of her diarrhea reduced to 3-4 times per day, and the consistency became semi-solid. The dose of her loperamide was reduced. Home oxygen evaluation revealed a saturation of 94% on room air at rest and a saturation of 93% with exertion on room air. Her serum albumin levels remained around 3 mg/dL and serum calcium levels remained around 8 mg/dL (corrected calcium was within normal range). She was discharged without supplemental oxygen with advice to follow up with her oncologist in two weeks to discuss therapy for her triple negative metastatic breast cancer.

Regarding her history of CLL, she recalls presenting with a singular lymph node on her right neck 20 years ago. She underwent an excisional biopsy and was diagnosed with CLL. She reported no systemic treatment for her CLL and chose to treat herself with homeopathic remedies. Her complete blood count had been unremarkable for the past decade. Two years ago, on repeat CLL workup, flow cytometry identified an abnormal B cell population with CLL immunophenotype, but she did not meet the definition criteria for CLL based on the absolute numbers of the lymphocytes. Cytogenetics detected 13q deletion in 20.5% of cells (associated with the best prognosis) with no high risk peculiarities (11q deletion / 17p deletion / Trisomy 12 / CCND1-IGH fusion were not detected). She was explained that CLL is an indolent hematologic malignancy that does not always require therapy and that in the absence of symptoms, it can be managed with observational surveillance. Two explanations were put forth - she either had monoclonal B-cell lymphocytosis (MBL) and that she never had CLL, or she had CLL but the therapies that she received in the past for her breast cancer had an effect on the CLL cells leading to a decrease of the lymphocytosis. She was also explained that MBL can transform to CLL at approximately 1% per year.

## Discussion

The most common adverse effects of capecitabine include myelosuppression (most commonly lymphopenia), nausea, vomiting, constipation, diarrhea, stomatitis, fatigue, hand-foot syndrome (palmar-plantar erythrodysesthesia​​), liver function abnormalities (most commonly hyperbilirubinemia) and rare cardiotoxicity (ischemia). While GI effects, alopecia, dermatitis, and neutropenia are more common with intravenous 5-fluorouracil, hand-foot syndrome (HFS) occurs more often during capecitabine treatment [6]. A history of fluorouracil hypersensitivity, severe renal impairment, severe hepatic impairment, dihydropyrimidine dehydrogenase deficiency, or severe leukopenia, neutropenia, or thrombocytopenia are all contraindications. Severe GI adverse effects of capecitabine include enterocolitis and rarely ischaemic colitis, which may require surgical intervention (emergency laparotomy and partial enterectomy) [7, 8]. 

Pseudomembranous colitis, which is usually caused by toxins produced from *Clostridium difficile*, usually presents in patients with a recent history of antibiotic use and more rarely occurs post-chemotherapy. Common chemotherapies associated with *Clostridium difficile *associated diarrhea (CDAD) include 5-fluorouracil [9], docetaxel [10], paclitaxel [11] and carboplatin [12]. Complications include dehydration, electrolyte imbalance, prerenal acute kidney failure (due to hypovolemia), toxic megacolon, and bowel perforation. 20% to 35% of cancer patients have relapse or reinfection after their first episode of disease [13, 14]. The median time to resolution of diarrhea is delayed and the cure rate is lower among oncology patients compared with those without cancer. Because these patients have many explanations for diarrhea, one of the greatest clinical problems in managing cancer patients with positive *Clostridium difficile *testing is establishing whether the positive results represent colonization or actual disease. Colonoscopy with biopsy has been indicated as a standardized diagnostic measure in patients on capecitabine with diarrhea refractory to conservative methods [15]. Capecitabine-associated ileitis tends to localize in the terminal ileum [16] with a granular erythematous appearance of mucosa with ulcerations. Eosinophilic infiltrates with the development of pseudomembrane from inflammatory exudate have been reported. Our patient also had a history of tetracycline hypersensitivity and had a history of high rates of adverse effects with multiple medications. This might put her at a higher risk for capecitabine-induced colitis and even *Clostridium difficile*-associated diarrhea (CDAD) as compared with other patients with no known drug hypersensitivities. 

In a 2013 study involving two double-blind trials that randomly allocated 1,105 patients with CDAD to fidaxomicin or vancomycin treatment, Cornely and colleagues reported better response and less chance of recurrence with fidaxomicin in cancer patients [time to resolution of diarrhea (TTROD) was longer for patients with cancer on vancomycin therapy, but the TTROD was similar for all types of patients on fidaxomicin therapy] [17]. The observation made by Taur and colleagues that metronidazole use is associated with Enterococcal predominance in the stool and a nine-fold increased rate of vancomycin-resistant *Enterococcus *bacteremia casts doubt on its usefulness in the management of CDAD [18]. Vancomycin and fidaxomicin may be better because of their relatively narrow spectrum of action.

Other causes of colitis in cancer patients include neutropenic colitis (due to myelotoxicity), radiation enteritis (due to past history of abdominal or whole body therapeutic radiation exposure), infectious colitis (bacterial - *Campylobacter jejuni*, Salmonella, Shigella, *Escherichia coli*, *Yersinia enterocolitica*, Mycobacterium tuberculosis; viral - Norovirus, Rotavirus, Adenovirus, Cytomegalovirus, parasitic - *Entamoeba histolytica*), or colonic graft-versus-host-disease (GVHD - occurring after stem cell transplants). Our patient had an unremarkable complete blood count (CBC) with no signs of neutropenia, and no history of abdominal radiation exposure or stem cell transplants. In her case, gastrointestinal biofire panel was not sent as a CT scan guided the investigation towards possible *Clostridium difficile *infection, which turned out to be positive.

Our patient also had a history of bilateral pleural effusions and third spacing from docetaxel therapy. However, she continued to have mild-to-moderate pleural effusions after discontinuing docetaxel and after starting capecitabine. Malignant pleural effusions were ruled out via thoracentesis and cytology. By her fifth day of hospitalization, her diarrhea had progressed into severe protein-losing enteropathy, and the resultant hypoalbuminemia further exacerbated her extracellular fluid extravasation. While docetaxel is notorious for causing pleural effusions, capecitabine is not usually known for this adverse effect [19]. 

## Conclusions

Palliative capecitabine therapy has been commonly used for triple-negative breast cancer management. There are numerous common side effects with dermatological and gastrointestinal ones being the most common. It has been associated with terminal ileitis with negative infective serology on multiple occasions. Clinically, it becomes challenging to accurately attribute the cause of a colitis-like presentation in the context of a positive *Clostridium difficile *serology. Abdominal CT and colonoscopy are vital. Early initiation of fidaxomicin therapy may produce superior outcomes. Risk of capecitabine-associated third spacing might not be exclusively limited to hypoalbuminemia, and may reflect intrinsic tendency to cause capillary leakage similar to docetaxel. However, more studies are needed for corroboration.
